# Is Hashimoto thyroiditis associated with increasing risk of thyroid malignancies? A systematic review and meta-analysis

**DOI:** 10.1186/s13044-021-00117-x

**Published:** 2021-12-03

**Authors:** Pouya Abbasgholizadeh, Amirreza Naseri, Ehsan Nasiri, Vahideh Sadra

**Affiliations:** 1 Student Research Committee, Tabriz University of Medical Sciences, Tabriz, Iran; 2 Aging Research Institute, Tabriz University of Medical Sciences, Tabriz, Iran; 3 Research Center for Evidence-Based Medicine, Iranian EBM Centre: A Joanna Briggs Institute (JBI) Center of Excellence, Tabriz University of Medical Sciences, Tabriz, Iran; 4 Endocrine Research Center, Tabriz University of Medical Sciences, Golgasht street, Tabriz, Iran

**Keywords:** Hashimoto Thyroiditis, Chronic autoimmune thyroiditis, Thyroid neoplasms, Systematic review, Meta-analysis

## Abstract

**Background and purpose:**

Hashimoto thyroiditis (HT) is the most common inflammatory autoimmune thyroid disease and also the most common cause of hypothyroidism in developed countries. There is evidence of the role of HT in developing thyroid cancers (TCs). This study investigated the association between HT and different types of TCs.

**Methods:**

Results of a comprehensive search in three major databases, as well as hand searching, were screened in title/abstract and full-text stages and the relevant data were extracted from the studies that met the inclusion criteria. Risk of bias (RoB) was assessed using the Joanna Briggs Institute (JBI) critical appraisal tools and the meta-analysis was conducted with Comprehensive Meta-Analysis software.

**Results:**

Out of 4785 records, 50 studies were included in the systematic review, and 27 of them met the criteria for quantitative synthesis. The results indicated a significant role for HT in developing papillary TC (OR: 1.65; 95% CI: 1.04 to 2.61), medullary TC (OR: 2.70; 95% CI: 1.20 to 6.07) and lymphoma (OR:12.92; 95% CI: 2.15 to 77.63); but not anaplastic TC (OR: 1.92; 95% CI: 0.29 to 1.90) and follicular TC (OR: 0.73; 95% CI: 0.41 to 1.27). Also, this study found a significant association between HT and thyroid malignancies (OR: 1.36; 95% CI: 1.05 to 1.77).

**Conclusion:**

Although we found a significant association between HT and some types of TCs, High RoB studies, high level of heterogeneity, and the limited number of well-designed prospective studies, suggested the need for more studies to reach more reliable evidence.

**Supplementary Information:**

The online version contains supplementary material available at 10.1186/s13044-021-00117-x.

## Introduction

Chronic lymphocytic thyroiditis also called “Hashimoto thyroiditis” (HT) is the most common inflammatory autoimmune thyroid disease and the most common cause of hypothyroidism in regions with adequate amounts of iodine [1]. HT was first delineated by Japanese surgeon Hakaru Hashimoto as an autoimmune disease [2]. HT is characterized by immune cells infiltration of the thyroid gland as a result of failure in immune tolerance. This condition frequently affects females (more than 10:1 ratio of females to males) [3]. The occurrence of HT has increased during the last decades. Thyroid cancer (TC) is the most common endocrine tumor and the occurrence of TC has increased rapidly worldwide. Papillary thyroid carcinoma (PTC) is the most common type of thyroid neoplasms and accounts for 80-90% of all thyroid cancers. It occurs more frequently in females.

Rudolf Virchow first described the link between chronic inflammation and cancer in 1893, which is now well determined [4]. The association between HT and PTC was first described by Dailey et al. in 1955 [5]. Despite several retrospective and prospective studies performed, the relationship between them remains controversial. A recent meta-analysis of 64,628 patients in 36 studies reported a relation between HT and PTC and an association between HT and thyroid lymphoma [6]. Consistently with this finding, several studies have been performed and they reported that HT is associated with a greater probability of developing PTC [7]. Another meta-analysis revealed the correlation between HT and PTC and this systematic review only investigated the incidence of HT in TC patients and not the incidence of TC in HT patients [8]. In contrast with this finding, Jankovic et al. reported no significant association between HT and TC based on 8 fine-needle aspiration studies [9].

Given the selection bias and limitations of previous studies as well as new publications in this area, an updated systematic review is needed to better clarify the association between HT and TC. Therefore, we elaborated a new meta-analysis via a complete investigation of the literature aiming to evaluate the association between HT and TCs, and also the investigation of the role of HT in different subgroups of TC, based on current knowledge.

## Methods

This systematic review was conducted following the Preferred Reporting Items for Systematic Reviews and Meta-Analyses (PRISMA) statement [10].

### Search

After getting the approval of the study protocol, an electronic search was conducted in 3 major databases including Medline via PubMed, EMBASE, and Scopus, with ((Chronic autoimmune thyroiditis) OR Hashimoto) AND (thyroid neoplasm* OR thyroid carcinoma* OR thyroid cancer* OR thyroid adenoma* OR thyroid malignanc*) and related MeSH keywords on 23 February 2021.

### Study selection

Results of the electronic search were imported into EndNote 20 and after removing the duplicated studies, the remaining records were screened in two title/abstract and full-text stages. Two independent authors screened the studies and in case of any disagreements, a third author deemed the issue. For full coverage of any published studies, after selecting the final articles to be included in this systematic review, the reference lists of these articles and recently published reviews have been checked for possible inclusion in our study.

### Eligibility criteria

We included the journal articles which assess the possible relation between HT and TC with both retrospective and prospective study designs. In case of lack of a control group, the study was included in our systematic review but excluded from the meta-analysis. We only selected the articles which have been written in English and animal studies, case reports, review articles, editorials, letters, conference abstracts, and withdrawn articles were excluded from our study.

### Data extraction

Data extraction was conducted by two authors with an electronic table in Microsoft Word. The following data were extracted from each study: the name of the first author of the study, the year of study publication, the study design which could be retrospective or prospective, the setting of the study, the method for diagnosis of thyroid cancer, the sample size, the mean and standard deviation of ages, the number of female and male cases, the type of thyroid cancer and finally the rate of TC between HT cases and control group, or rate of HT between TC and control group.

### Risk of Bias assessment

The risk of bias (RoB) in included studies was assessed using the Joanna Briggs Institute (JBI) critical appraisal tools for cohort or case-control studies based on the study design [11]. The checklist for case-control studies includes 10 questions and the cohort studies’ checklist includes 11 questions. These tools assess the similarity of case and control groups, using a standard and similar method for assessing the condition, appropriate dealing with cofounding factors, enough period of interest, and appropriate statistical analysis.

### Statistics

All the statistical analyses were conducted using the second version of Comprehensive Meta-Analysis (CMA.2) software with 95% confidence intervals and a 0.05 level of significance. I^2^ model was used for assessing the heterogeneity between the studies, and for outcomes with more than 50% level of heterogeneity, a random effect model was used. The number of events in case and control groups in both study designs and also the size of the group was imported into the CMA and the odds ratio (OR) was collected for each study. Then the results were then combined in both random and fixed effect models and the ORs for each subgroup (based on the type of thyroid cancer) as well as the overall result were calculated and presented by forest plot.

## Results

**Fig. 1:**
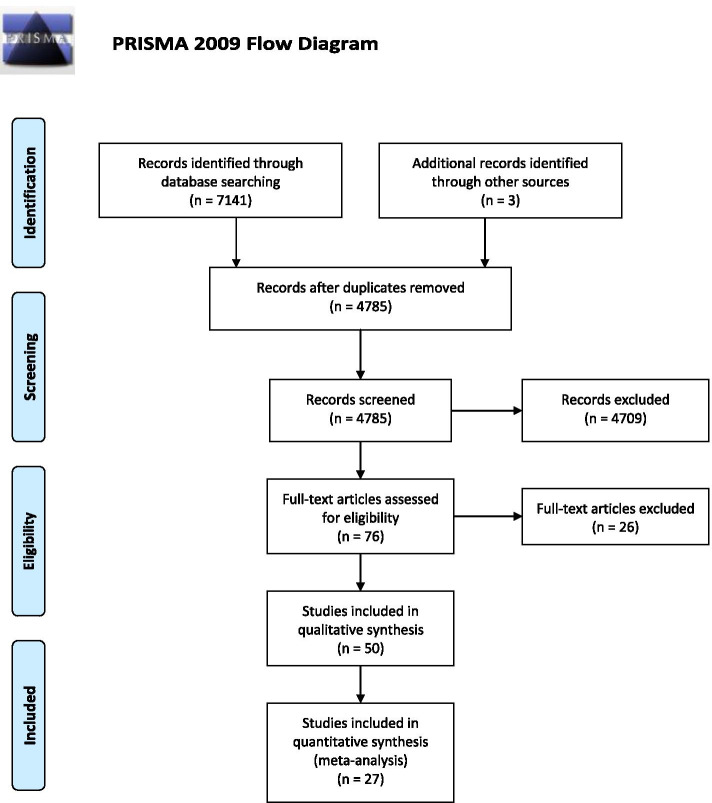
PRISMA flow diagram

**Fig. 2:**
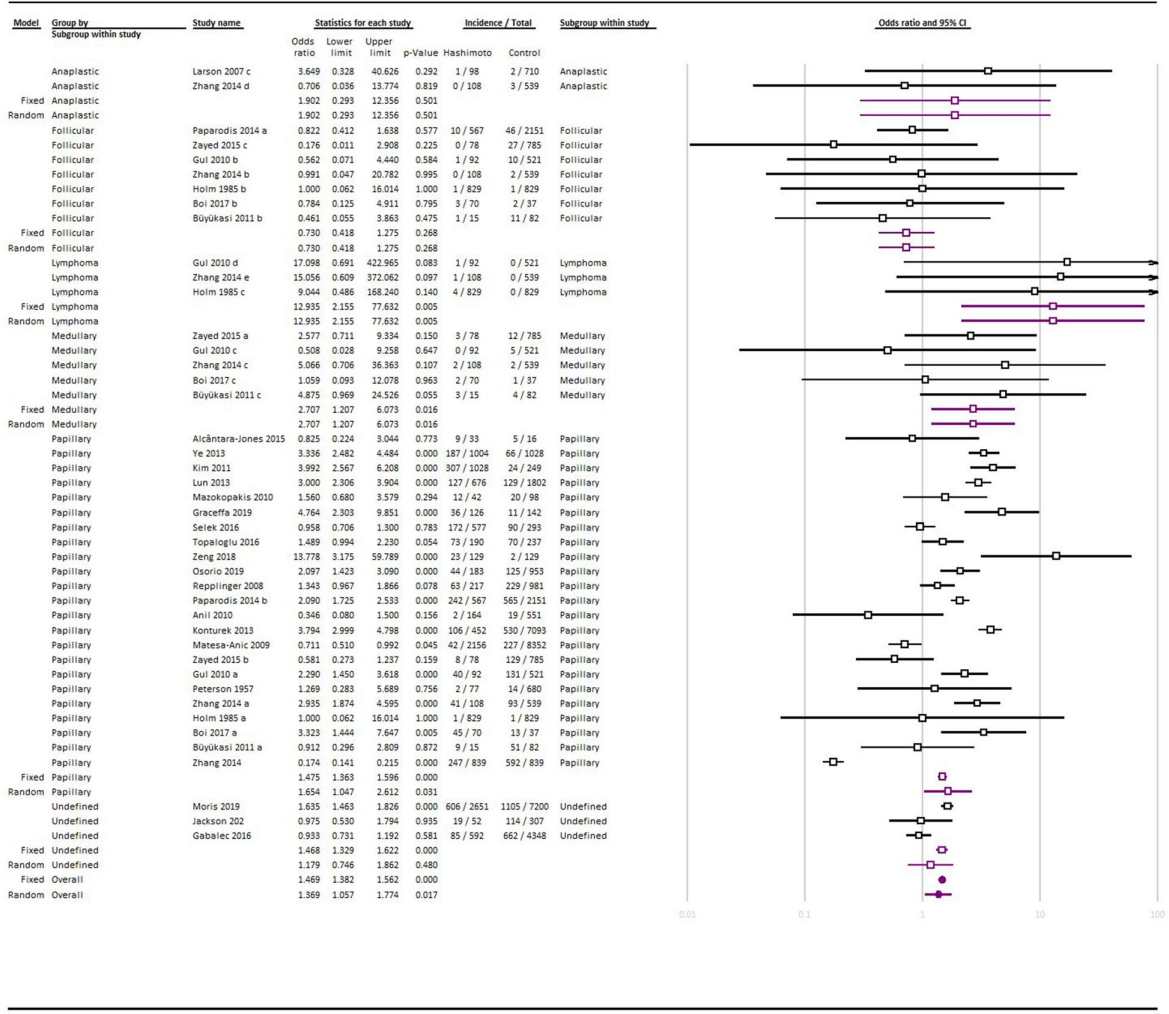
forest plot of the studies included in the meta-analysis

Globally, 7141 records were identified through database searching, and after removing the duplicated studies, 4785 studies were screened. Finally, 50 studies were selected for qualitative synthesis and 27 of them, were included in the quantitative synthesis (Fig. 1). Among them, 23 studies found the rate of HT in TC cases; on the other hand, 29 of them assessed the rate of TC in HT cases, whereas 3 of them reported both of these findings. The characteristics of the included studies are summarized in Table 1. Figure 2 summarized the results of the meta-analysis.

**Table 1:** characteristics and summary of findings in the included studies

Study	Study design	Setting	Study method	Sample size	Mean age ± SD	Female: Male	Thyroid cancer type	Rate of Hashimoto’s thyroiditis (in percent)	
								Thyroid cancer	Control group
Alcântara-Jones 2015 [12]	Retrospective	Brazil	Thyroidectomy	49	48.5	30:3	Papillary	27.27 (9/33)	31.25 (5/16)
Zeng 2016 [13]	Retrospective	China	Thyroidectomy	619	45.9	484:135	Papillary	35.86 (222/619)	
Campos 2012 [1]	Retrospective	Brazil	Thyroidectomy	315	44.9	34:7	Papillary	26.83 (11/41)	1.12 (3/268)
Ye 2013 [14]	Retrospective	China	Thyroidectomy	2052	–	828:176	Papillary	18.63 (187/1004)	6.42 (66/1028)
Cipolla 2005 [15]	Retrospective	Italy	Thyroidectomy	178	–	68:21	Papillary	26.76 (19/71)	
Kim 2011 [16]	Retrospective	Korea	Thyroidectomy	1329	47.5	821:207	Papillary	29.86 (307/1028)	9.64 (24/249)
						32:20	Follicular	9.62 (5/52)	
Ahn 2011 [17]	Retrospective	Korea	Thyroidectomy	303	42.8	225:44	Papillary	21.56 (58/269)	
Huang 2011 [18]	Retrospective	China	Thyroidectomy	1997	39.9	1450:338	Papillary	4.75 (85/1788)	
						153:56	Follicular	3.83 (8/209)	
Lun 2013 [19]	Retrospective	China	Thyroidectomy	2478	41.3	538:138	Papillary	18.79 (127/676)	7.16 (129/1802)
Moshynska 2008 [20]	Retrospective	Canada	Thyroidectomy	20	–	–	Lymphoma	60 (12/20)	
Singh 1999 [21]	Retrospective	United States	Thyroidectomy	453	41	267:121	Papillary	14.69 (57/388)	
						–	Follicular	2.08 (1/48)	
						–	Lymphoma	5.88 (1/17)	
Zhang 2014 [22]	Retrospective	China	Thyroidectomy	8524	43.1	–	Papillary	28.46 (592/2080)	
Nemetz 2011 [23]	Retrospective	Brazil	Thyroidectomy	52	51.3	48:4	Papillary	32.69 (17/52)	
Jeong 2012 [24]	Retrospective	Korea	Thyroidectomy	1357	44.5	1176:181	Papillary	26.46 (359/1357)	
Kashima 1998 [25]	Retrospective	Japan	Thyroidectomy	1533	42.6	1402:131	Papillary	18.33 (281/1533)	
Kebebew 2001 [26]	Retrospective	United States	Thyroidectomy	136	45.5	95:41	Papillary	30.15 (41/136)	
Yoon 2012 [27]	Retrospective	Korea	Thyroidectomy	195	45.9	166:29	Papillary	28.72 (56/195)	
Graceffa 2019 [28]	Retrospective	Italy	Thyroidectomy	305	50.6	258:47	Papillary	28.6 (36/126)	7.7 (11/142)
Selek 2016 [29]	Retrospective	Turkey	Thyroidectomy	870	47 ± 12		Papillary	30 (172/ 577)	31 (90/ 293)
Topaloglu 2016 [30]	Retrospective	Turkey	Thyroidectomy	427	Malignant: 49.10 ± 12.23 Benign: 47.78 ± 12.39	341:86	Papillary	38.4 (73/190)	29.5 (70/237)
Zeng 2018 [31]	Retrospective	China	Thyroidectomy	258	17.31 ± 3.21	212:46	Papillary	17.8 (23/129)	1.6 (2/129)
Osorio 2019 [7]	Retrospective	Colombia	Thyroidectomy	1136	47.5 ± 14.3	1047: 89	Papillary	24 (44/183)	13.11 (125/953)
Youssef Mohamed 2020 [32]	Retrospective	Egypt	Thyroidectomy	80	–	22: 58	Papillary	20 16/80	
JNawarathna 2018 [33]	Retrospective	Sri Lanka	Thyroidectomy	684	48 ± 12.5	611: 73	Papillary	OR: 0.867 (0.25-2.99)	
							Follicular	OR: 1.02 (0.22-4.58)	
								**Rate of thyroid cancer (in percent)**	
								**Hashimoto’s thyroiditis**	**Control group**
Repplinger 2008 [34]	Retrospective	United States	Thyroidectomy	1198		215:77	Papillary	29.03 (63/217)	23.34 (229/981)
Paparodis 2014 [35]	Retrospective	United States	Thyroidectomy	2718			Papillary	42.68 (242/567)	26.27 (565/2151)
							Follicular	1.76 (10/567)	2.14 (46/2151)
Anil 2010 [36]	Prospective	Turkey	FNA	715			Papillary	1.22 (2/164)	3.45 (19/551)
Konturek 2013 [37]	Retrospective	Poland	Thyroidectomy	7545	53.5		Papillary	23.45 (106/452)	7.47 (530/7093)
Mukasa 2011 [38]	Retrospective	Japan	FNA	2036			Papillary	1.77 (36/2036)	
							Lymphoma	0.10 (2/2036)	
Matesa-Anic 2009 [39]	Retrospective	Croatia	FNA	10,508	50	236:33	Papillary	1.95 (42/2156)	2.72 (227/8352)
Dailey 1955 [5]	Prospective	United States	Thyroidectomy	2336	37.5		Papillary	10.43 (29/278)	
Larson 2007 [40]	Retrospective	United States	Thyroidectomy	812	41	142:37	Papillary	34.7 (34/98)	20.4 (145/710)
						16:5	Follicular	9.2 (9/98)	1.69 (12/710)
						3:0	Anaplastic	1.02 (1/98)	0.28 (2/710)
Zayed 2015 [41]	Retrospective	Jordan	Thyroidectomy	180	51.3	9:6	Medullary	3.85 (3/78)	1.53 (12/785)
						102:35	Papillary	10.26 (8/78)	16.43 (129/785)
						18:9	Follicular	0 (0/78)	3.44 (27/785)
Gul 2010 [42]	Retrospective	Turkey	Thyroidectomy	613	43		Papillary	43.48 (40/92)	25.14 (131/521)
							Follicular	1.09 (1/92)	1.92 (10/521)
							Medullary	0 (0/92)	0.96 (5/521)
							Lymphoma	1.09 (1/92)	0 (0/521)
Mazokopakis 2010 [43]	Retrospective	Greece	Thyroidectomy	140	49.3	25:7	Papillary	28.57 (12/42)	20.41 (20/98)
Sclafani 1993 [44]	Retrospective	United States	Thyroidectomy	48	51.7		Papillary	12.5 (6/48)	
Peterson 1957 [45]	Retrospective	United States	Thyroidectomy	757			Papillary	2.60 (2/77)	2.06 (14/680)
Zhang 2014 [46]	Retrospective	China	Thyroidectomy	647	43.3		Papillary	37.96 (41/108)	17.25 (93/539)
							Follicular	0 (0/108)	0.37 (2/539)
							Medullary	1.85 (2/108)	0.37 (2/539)
							Anaplastic	0 (0/108)	0.56 (3/539)
							Lymphoma	0.93 (1/108)	0 (0/539)
Holm 1985 [47]	Prospective	Sweden	FNA	1656			Papillary	0.12 (1/829)	0.12 (1/829)
							Follicular	0.12 (1/829)	0.12 (1/829)
							Lymphoma	0.48 (4/829)	0 (0/829)
Moris 2019 [48]	prospective	United States	Thyroidectomy	9851	52.2 ± 15	8263: 1588	Undefined	22.8 (606/2651)	15.4 (1105/7200)
			FNA				Undefined	7.3 (284/3895)	4.7 (473/10168)
Jackson 2020 [49]	retrospective	United States	Thyroidectomy	359			Incidental thyroid cancer	15 (8/52)	10 (31/307)
							Thyroid cancer	37 (19/52)	37 (114/307)
Keskin 2016 [50]	prospective	Turkey	FNA and Thyroidectomy	300	12.1 ± 3.1	238: 62	Papillary	0.66 (2/300)	
Liu2017 [51]	retrospective	China	Thyroidectomy	927	46 ± 0	706: 221	Papillary		
Radetti 2019 [52]	prospective	Italy	FNA	904	10.6 ± 3.2	709: 195	Papillary	1.1 (10/904)	
Won2018 [53]	retrospective	Korea	FNA	89	11.1 ± 3.7	76: 13	Papillary	7.9 (7/89)	
Boi 2017 [54]	retrospective	Italy	FNA	645				28.9 (44/152)	7.4 (12/161)
			Thyroidectomy				Papillary	64.3 (45/70)	35.1 (13/37)
			Thyroidectomy				Follicular	4.3 (3/70)	5.4 (2/37)
			Thyroidectomy				Medullary	2.8 (2/70)	2.7 (1/37)
Gabalec 2016 [55]	retrospective	Czech republic	Thyroidectomy	4947			Undefined	29.5 (26/88)	15.2 (231/1515)
			FNA					14.2 (85/592)	15.2 (662/4348)
Büyükaşi 2011 [56]	retrospective	Turkey	Thyroidectomy	917	adult	743:174	All Cancer Types	19.4 (15/77)	9.8 (82/840)
							Papillary	60.0 (9/15)	63.41 (52/82)
							Follicular	6.6 (1/15)	13.41 (11/82)
							Medullary	20.0 (3/15)	4.87 (4/82)
Chen 2013 [57]	prospective	Taiwan		7605	adult	6845:755		1.58	0.14
Cipolla 2005 [15]	Retrospective	Italy	Thyroidectomy	178	–	68:21	Papillary	27.6 (13/47)	
Zhang 2014 [22]	Retrospective	China	Thyroidectomy	8524	43.1	–	Papillary	14.24 (247/839)	70 (592/839)
Graceffa 2019 [28]	Retrospective	Italy	Thyroidectomy	305	50.6	258:47	Papillary	28.83 (47/163)	
							Follicular	1.84 (3/163)	
							Medullary	0.61 (1/163)	
Uhliarova 2017 [58]	prospective	Slovakia	Thyroidectomy	2117	11.1 ± 3.7	1738: 379	All cancer types	83.64%) (266/318)

### Papillary thyroid cancer

45 of the included studies investigated the possible relation between HT and PTC. In these studies, the rate of HT in PTC cases was ranged between 4.75 to 38.4%, whereas the rate of PTC in HT ranged between 0.12 to 64.3%. The meta-analysis of 23 studies with an appropriate control group, found 1.65 OR (95% CI: 1.04 to 2.61; I^2^ test for heterogeneity: 96.48%) and the difference between the groups was significant (*p* = 0.03).

### Follicular thyroid cancer

13 studies assessed the possible association between HT and follicular thyroid cancer (FTC). In these studies, the rate of HT in the FTC group ranged between 2.08 to 9.62% and the rate of FTC in HT ranged between 0 to 9.2%. The meta-analysis of the 7 studies that met the proper inclusion criteria, reached 0.73 OR (95% CI: 0.41 to 1.27; I^2^ test for heterogeneity: 0%) and the difference between the groups was not statistically significant (*p* = 0.26).

### Medullary thyroid cancer

The possible role of HT in developing medullary thyroid cancer (MTC) was investigated in 6 studies. All of these studies assessed the rate of MTC in the HT group and it ranged between 0 to 20%. The meta-analysis of 5 studies with an appropriate control group, reached 2.70 OR for this outcome (95% CI: 1.20 to 6.07; I^2^ test for heterogeneity: 0%) and the difference between the groups was significant (*p* = 0.01).

### Lymphoma

6 studies investigated the relation between HT and lymphoma and the rate of HT in the lymphoma group was 5.88 and 60% in two studies. The range of lymphoma in HT was between 0.1 to 1.09%. The meta-analysis of 3 studies concerning this outcome reached 12.93 OR (95% CI: 2.15 to 77.63; I^2^ test for heterogeneity: 0%) and the difference between these groups was significant (*p* = 0.01).

### Anaplastic thyroid cancer

Only two studies assessed the relation between anaplastic thyroid cancer (ATC) and HT and the rate of TC in the HT group was 0 and 1.02% in these studies. The meta-analysis reached 1.92 OR (95% CI: 1.90 to 0.29; I^2^ test for heterogeneity: 0%) and the difference between groups was not statistically significant (*p* = 0.05).

### All cancer types

Twenty seven studies had an appropriate control group which allowed us to calculate the OR and include them in the meta-analysis. The results showed 1.36 OR (95% CI: 1.05 to 1.77; I^2^ test for heterogeneity: 93.66%) and there was a significant difference between case and control groups in terms of incidence of TCs (*p* = 0.01).

### Risk of Bias

**Fig. 3:**
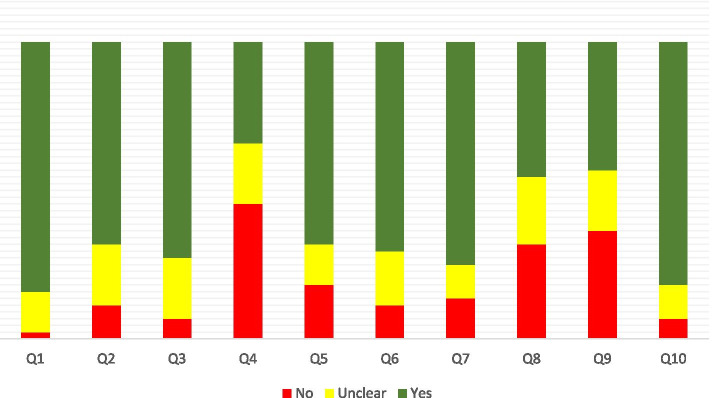
Risk of Bias in the studies based on Joanna Briggs Institute (JBI) checklist

The RoB assessment based on the JBI checklist is presented in Fig. 3. Based on our assessment, appropriately dealing with confounding factors was the most prevalent source of bias in included studies. The appropriate and complete follow-up period was the other source of bias in these studies. Generally, there is a concerning risk of bias in these studies which can affect these outcomes. The details of the RoB assessment are presented in Supplementary material 1.

## Discussion

This study investigated the possible relationship between HT and different types of TCs. The results indicated a significant correlation between HT and thyroid malignancies in particular with PTC, MTC, lymphoma but not with ATC and FTC. Also, this study found a significant association between HT and thyroid malignancies.

As the most common cause of hypothyroidism in developed countries [59], the role of HT in developing thyroid malignancies, should be considered by the clinicians. As fine needle aspiration (FNA) has poor accuracy in the diagnosis of TCs in patients with thyroiditis, diagnosis of TC in the presence of HT is challenging. Previous studies have found a better prognosis for TC in case of coexistence of HT, because of earlier diagnosis based on routine medical follow-up [18]. Moreover, a less aggressive form of malignancy in PTC patients in the top of HT has been reported, though but this conclusion was associated with controversies in an endemic area of iodine deficiency goiter [60].

Despite multiple hypotheses in this regard, the underlying mechanism of developing malignancies in HT patients is not fully understood [61, 62]. One of these mechanisms may rely on the inflammatory process in HT. Inflammatory reactions create free radical oxygen, resulting in DNA damage and mutations that finally cause the development of PTC [9]. Another hypothesis states that malignant transformation is caused by increased levels of TSH that stimulate thyroid tissue epithelial proliferation [61]. A recently published study assessed the prognostic value of FOXP3 in PTC and the difference in its expression in concomitant HT. FOXP3 is a PTC-related marker and its expression by HT infiltrating lymphocytes suggested a relationship between HT and PTC [32].

Despite the historical discussion about the possible role of HT in developing TCs, current guidelines didn’t accept HT as a risk factor for developing thyroid malignancies [63]. Some experts believe that a good prognosis of TCs and particularly PTC, as the most incident thyroid malignancy, leads to a decrease in allocation of resources toward designing and conducting well-designed studies to identify predictive factors and improving the management of outcomes [64]. The controversial outcomes of the studies highlighted a need for more prospective studies with appropriate control groups and considering the possible cofounding factors to reach more reliable evidence.

Our meta-analysis as the most reliable evidence in this regard found a significant association between HT and MTC based on 5 published studies. This finding is obtained based on a retrospective point of view and only 11 cases of MTC were reported in 526 investigated cases of HT in our included studies. MTC is the third most common TC that originates from the parafollicular cells with an unfavorable prognosis [65, 66]. Previously the reports of this relation were limited to case reports [67–72]. One of the suggested pathophysiological bases for this relation is the occurrence of HT in response to MTC, so future prospective studies can give better insight in this regard. Also, the results of Zayed et al. only found such an association only in female patients [41], which should be more investigated in future studies.

One of the limitations of this study was the high level of heterogeneity between different studies. These differences can arise from multiple sources. Differences in pathological interpretation of HT, genetic factors, diagnostic methods for thyroid malignancies including the FNA and total thyroidectomy can cause variations in the reported rate of coexistence of HT and TC in our included studies. Besides, the variation in OR can arise from differences in defining the control groups.

A comprehensive search in three major databases and the adding of hand searching results was one of the strengths of this study that led to full coverage of published studies that met our inclusion. Besides, carefully selecting and extracting the data, was the other strength of this systematic review. Unlike previous studies, we conducted our meta-analysis based on OR, therefore, the studies without an appropriate control group were excluded from our meta-analysis. This made the findings of our study more practical and obvious.

## Conclusion

Based on the current knowledge, HT is associated with developing thyroid malignancies, particularly PTC, MTC, lymphoma but not with ATC and FTC. Studies with high RoB, the high level of heterogeneity between different studies, and the limited number of well-designed prospective studies make the available evidence uncertain, so there is a need for more studies to reach more reliable conclusions.

## Supplementary Information

**Additional file 1.**

## Data Availability

Not applicable.

## Abbreviations

HT: Hashimoto thyroiditis
TC: Thyroid cancer
PTC: Papillary thyroid carcinoma
PRISMA: Preferred Reporting Items for Systematic Reviews and Meta-Analyses
RoB: Risk of bias
JBI: Joanna Briggs Institute
CMA: Comprehensive Meta-Analysis
OR: Odds ratio
FTC: Follicular thyroid cancer
MTC: Medullary thyroid cancer
ATC: Anaplastic thyroid cancer
