# Supplementary material for: Is Hashimoto thyroiditis associated with increasing risk of thyroid malignancies? A systematic review and meta-analysis
Source: Thyroid Res. 2021 Dec 3;14:26. doi: 10.1186/s13044-021-00117-x (PMC8641157; doi:10.1186/s13044-021-00117-x)
Supplement: Supplementary file 1 — Additional file 1. [file 13044_2021_117_MOESM1_ESM.docx]

| **Study** | **Question 1** | **Question**  **2** | **Question 3** | **Question**  **4** | **Question 5** | **Question 6** | **Question**  **7** | **Question**  **8** | **Question**  **9** | **Question**  **10** | **Question**  **11** |
| --- | --- | --- | --- | --- | --- | --- | --- | --- | --- | --- | --- |
| Graceffa2019 | YES | NO | YES | UNCLEAR | YES | UNCLEAR | NO | YES | NO | YES | N/A |
| Jackson2020 | YES | UNCLEAR | YES | NO | YES | NO | YES | NO | NO | YES | N/A |
| Liu2017 | YES | YES | YES | NO | YES | YES | YES | YES | UNCLEAR | YES | N/A |
| Selek2016 | UNCLEAR | YES | YES | NO | YES | YES | NO | YES | UNCLEAR | YES | N/A |
| Uhliarova2017 | YES | YES | NO | UNCLEAR | YES | UNCLEAR | YES | YES | YES | YES | N/A |
| Won2018 | YES | NO | UNCLEAR | NO | YES | NO | UNCLEAR | NO | YES | NO | N/A |
| Boi2017 | YES | NO | YES | UNCLEAR | YES | UNCLEAR | NO | YES | NO | UNCLEAR | N/A |
| Gabalec2016 | UNCLEAR | YES | YES | NO | YES | YES | YES | YES | YES | YES | N/A |
| ZENG2018 | YES | YES | NO | NO | UNCLEAR | YES | YES | UNCLEAR | YES | YES | N/A |
| Osorio2019 | UNCLEAR | UNCLEAR | UNCLEAR | NO | YES | UNCLEAR | YES | UNCLEAR | YES | UNCLEAR | N/A |
| JNawarathna2018 | YES | YES | NO | NO | YES | YES | YES | YES | UNCLEAR | NO | N/A |
| Zhang2014 | YES | NO | YES | YES | YES | YES | UNCLEAR | NO | UNCLEAR | YES | N/A |
| Peterson1957 | YES | YES | YES | YES | NO | YES | YES | YES | YES | YES | N/A |
| Sclafani1993 | UNCLEAR | YES | YES | UNCLEAR | UNCLEAR | NO | YES | YES | NO | YES | N/A |
| Mazokopakis2010 | YES | UNCLEAR | YES | YES | YES | YES | YES | UNCLEAR | YES | YES | N/A |
| Gul2010 | YES | NO | YES | YES | YES | UNCLEAR | YES | YES | UNCLEAR | NO | N/A |
| Zayed2015 | YES | YES | UNCLEAR | NO | YES | YES | NO | YES | NO | UNCLEAR | N/A |
| Larson2007 | YES | YES | YES | UNCLEAR | YES | NO | UNCLEAR | YES | NO | YES | N/A |
| de Alcantara-Jones2015 | Unclear | Yes | yes | Yes | Yes | Yes | Yes | No | No | yes | N/A |
| Zeng2016 | Yes | Yes | Unclear | Unclear | Yes | Yes | No | No | No | Yes | N/A |
| Campos2012 | Yes | Unclear | unclear | Yes | Yes | No | Unclear | No | Unclear | Unclear | N/A |
| Ye2013 | Unclear | Yes | Yes | Yes | Yes | Yes | Yes | No | Unclear | Yes | N/A |
| Kim2011 | Yes | Yes | Yes | unclear | No | Yes | Yes | No | Yes | Yes | N/A |
| Ahn2011 | Yes | Yes | Yes | No | Yes | yes | yes | Unclear | Yes | Yes | N/A |
| Huang2011 | Yes | Yes | Yes | No | No | Yes | Unclear | Unclear | Unclear | Yes | N/A |
| Lun2013 | Yes | Unclear | Yes | Unclear | No | Yes | Yes | unclear | No | Yes | N/A |
| Moshynska2008 | No | Unclear | Unclear | No | Unclear | unclear | No | No | No | Unclear | N/A |
| Singh1999 | Yes | Yes | Yes | Unclear | Unclear | Yes | Yes | Unclear | Unclear | Yes | N/A |
| Zhang2014 | Yes | Yes | Yes | Yes | Yes | Yes | Yes | yes | Yes | Yes | N/A |
| Nemetz2011 | Yes | Yes | Yes | No | Unclear | Yes | Yes | Yes | No | Yes | N/A |
| Jeong2012 | Yes | Yes | Yes | Yes | Yes | Yes | Yes | Yes | Yes | Yes | N/A |
| Kashima1998 | Yes | Yes | Yes | No | No | Yes | Yes | Unclear | Yes | Yes | N/A |
| Kebebew2001 | Yes | Yes | Yes | Yes | Yes | Yes | Yes | Yes | Yes | Yes | N/A |
| Yoon2012 | Yes | Yes | Yes | No | unclear | Unclear | Yes | No | No | Yes | N/A |
| Repplinger2008 | Yes | Unclear | Unclear | No | No | Yes | Yes | No | No | Yes | N/A |
| Paparodis2014 | Yes | Yes | Yes | Yes | Yes | Yes | Yes | Yes | Yes | Yes | N/A |
| Anil2010 | Yes | Yes | Yes | No | No | Yes | Yes | Unclear | Yes | Yes | N/A |
| Cipolla2005 | Yes | Yes | Yes | Yes | Yes | Yes | Yes | Yes | Yes | Yes | N/A |
| Konturek2013 | Yes | Yes | Yes | No | yes | Unclear | Yes | No | No | Yes | N/A |
| Mukasa2011 | Yes | Unclear | Unclear | No | yes | Yes | Yes | No | No | Yes | N/A |
| Matesa-Anic2009 | Yes | Yes | unclear | Yes | Yes | Yes | Yes | Yes | Yes | Yes | N/A |
| Dailey1955 | Yes | Yes | Yes | No | Yes | yes | yes | Unclear | Yes | Yes | N/A |
| Chen 2013 | yes | unclear | yes | yes | no | yes | yes | no | no | yes | N/A |
| Youssef Mohamed 2020 | Yes | Yes | Yes | Yes | Yes | Yes | Yes | Yes | Unclear | Yes | N/A |
| Silva de Moris 2019 | NO | UNCLEAR | YES | NOT APPLICABLE | UNCLEAR | UNCLEAR | NO | YES | NO | UNCLEAR | YES |
| Keskin2016 | YES | YES | UNCLEAR | UNCLEAR | YES | NO | YES | YES | UNCLEAR | NO | UNCLEAR |
| Radetti2019 | YES | UNCLEAR | YES | YES | YES | YES | YES | UNCLEAR | YES | YES | YES |
| Holm1985 | YES | UNCLEAR | YES | UNCLEAR | UNCLEAR | UNCLEAR | YES | YES | NO | UNCLEAR | YES |
| Oktay Büyükaşık 2011 | YES | UNCLEAR | NO | YES | YES | YES | NO | UNCLEAR | YES | YES | UNCLEAR |

Table 2 – results of risk of bias assessment.
